# Robotic ureteral reconstruction for endometriosis-induced strictures: insights from a multi-institutional experience

**DOI:** 10.1007/s00345-025-05982-x

**Published:** 2025-10-04

**Authors:** Matthew Lee, Sonam Saxena, Kelley Zhao, Cameron Dodd, Randall Lee, Michael Stifelman, Lee Zhao, Daniel D. Eun

**Affiliations:** 1 Department of Urology, Lewis Katz School of Medicine at Temple University, 255 S 17th Street, Medical Tower 7th Floor, Philadelphia, PA 19103 USA; 2 Department of Urology, Hackensack Meridian School of Medicine, Nutley, NJ USA; 3 Department of Urology, New York University Grossman School of Medicine at New York University Langone Medical Center, New York, NY USA; 4 Department of Urology, Sidney Kimmel Medical College at Thomas Jefferson University, Philadelphia, PA USA

**Keywords:** Robotics, Endometriosis, Ureter stricture, Reconstruction

## Abstract

**Purpose:**

To investigate outcomes of robotic ureteral reconstruction (RUR) in female patients with ureteral strictures caused by peri-ureteral endometriosis lesions.

**Methods:**

We retrospectively reviewed our multi-institutional Collaborative of Reconstructive Robotic Ureteral Surgery (CORRUS) database to identify all consecutive patients undergoing RUR for surgical management of endometriosis-induced ureteral strictures between 2017 and 2022. Indications for surgery included female patients with radiographic evidence of ureteral strictures and/or decreasing renal function on renal scan. We performed a descriptives analysis of perioperative outcomes in patients who met inclusion criteria. Surgical success was defined as freedom from additional interventions for recurrent ureteral stenosis.

**Results:**

Overall, 19 patients met the inclusion criteria. Median age was 39 (IQR 30–43) years. Ureteral strictures were located in the middle ureter in 4 (21.1%) patients and in the distal ureter in 15 (78.9%) patients. Fourteen (73.6%) patients had a known preoperative diagnosis of endometriosis. RUR techniques included refluxing reimplantation (47.4%), side-to-side reimplantation (21.1%), ureteroureterostomy (21.1%), and buccal mucosa graft ureteroplasty (10.5%). There was one (5.3%) major postoperative complication (Clavien > 2) in which a patient developed an intrabdominal abscess requiring drainage by interventional radiology. Five (26.3%) patients were ultimately diagnosed postoperatively with endometriosis based on surgical pathology. At a median follow-up of 22.5 (IQR 11.7–41.5) months, 18 (94.7%) patients were surgically successful.

**Conclusion:**

Clinicians should maintain a high index of suspicion for endometriosis in premenopausal women with ureteral stricture disease. RUR techniques may be effective for the management of patients with ureteral strictures secondary to endometriosis.

## Introduction

Ureteral involvement in endometriosis is an uncommon but clinically significant manifestation of the disease, occurring in approximately 1% of women with endometriosis [1–3]. Management of ureteral strictures caused by endometriosis can be challenging, with endoscopic options such as balloon dilation or laser ureterotomy demonstrating limited long-term success rates [4, 5]. Surgical reconstruction is often required for definitive treatment and typically involves an extensive dissection to free the ureter from surrounding fibrotic and endometriotic tissue.

The existing literature has largely focused on ureterolysis as the primary surgical approach for ureteral endometriosis [6–10]. However, in cases involving large, densely adherent or deeply infiltrative endometriotic lesions, ureterolysis alone is often not adequate or feasible [11, 12]. In these complex situations, definitive ureteral reconstruction approaches should be considered. We present a multi-institutional series on outcomes of robotic ureteral reconstruction (RUR) for complex endometriosis-induced ureteral strictures.

## Methods and materials

We retrospectively reviewed our multi-institutional Collaborative of Reconstructive Robotic Ureteral Surgery (CORRUS) database to identify all patients undergoing RUR for surgical management of previously recognized or unrecognized endometriosis-induced ureteral strictures between January 2017 and December 2022. Surgical procedures were performed across three institutions using the single port or multiport da Vinci Surgical System (Intuitive Surgical, Sunnyvale, California). Indications for surgical intervention included female patients with cross sectional radiographic imaging demonstrating a ureteral stricture and/or decreasing ipsilateral split function and/or prolonged half times (> 20 min) on a nuclear medicine renal scan after furosemide administration. Both patients with a preoperative diagnosis of endometriosis and those in whom endometriosis was diagnosed on final surgical pathology were included in the analysis. Not all patients included in this study exhibited obstructive symptoms (pelvic or flank pain) or associated symptoms of endometriosis (dyspareunia, dysmenorrhea, menorrhagia, hematuria). Furthermore, our cohort included patients who did not undergo prior medical therapy for management of endometriosis. Final pathology of all intraoperative periureteral tissue specimens confirmed endometrial tissue.

The decision to perform a specific RUR technique was determined by the primary surgeon based on preoperative and intraoperative factors. For distal ureteral strictures caused by extensive circumferential compression from endometriotic tissue, a refluxing ureteral reimplantation was typically performed. No patients required adjunctive techniques, including a Boari flap or psoas hitch to facilitate a tension-free anastomosis. In cases where the stricture was located more proximally and a sufficient length of healthy distal ureter was preserved, a ureteroureterostomy was favored. In redo cases or when resection of the endometriotic lesion did not result in substantial periureteral damage, a non-transecting approach was preferred. In these scenarios, if the stricture involved the distal ureter, a side-to-side ureteral reimplantation was performed, and if involved the mid-to-distal ureter, a buccal mucosa graft onlay ureteroplasty was utilized. A double J ureteral stent was placed during each case. Intravenous and intraureteral indocyanine green under near-infrared fluorescence may be utilized to assess ureteral perfusion and help delineate ureteral anatomy, respectively. A ureteroscopy was not routinely performed in all patients and was reserved for cases in which ureteral identification was difficult.

Patients were typically evaluated with serial office visits at 3, 6 and 12 months postoperatively. The double J ureteral stent was generally removed between 4 and 6 weeks postoperatively. Postoperative imaging protocols varied across participating institutions. In general, patients underwent either a renal ultrasound or a diuretic renogram shortly following stent removal. Additional imaging, typically in the form of cross-sectional studies or repeat diuretic renogram was performed between 6 and 12 months postoperatively to assess for functional and anatomic outcomes. Surgical success was assessed at each postoperative visit and was defined as the absence of additional surgical interventions or indwelling hardware for recurrent ureteral stenosis.

## Results

Our cohort included 19 patients who underwent robotic ureteral reconstruction for endometriosis-induced strictures (Table 1). Median age of our cohort was 39 (IQR 30–43) years. Fourteen (73.6%) patients had a preoperative diagnosis of endometriosis. A majority of strictures were located in the distal ureter (78.9%), which was defined as strictures located beneath the lower sacroiliac joint border. All patients in this cohort had evidence of hydronephrosis secondary to the ureteral stricture. Two (10.5%) patients had chronic kidney disease (Stage ≥ 2). A total of 4 (21.1%) patients underwent prior failed endoscopic or surgical ureteral stricture intervention. Median operative time was 197.0 (IQR 154.0-217.0) minutes and estimated blood loss was 50.0 (IQR 37.5–55.0) milliliters. The median stricture length was 2.0 (IQR 2.0–3.0) centimeters. Various RUR techniques were performed. Refluxing reimplantation was performed in 9 (47.4%) patients, side-to-side reimplantation was performed in 4 (21.1%) patients, ureteroureterostomy was performed in 4 (21.1%) patients, and buccal mucosa graft onlay ureteroplasty was performed in 2 (10.5%) patients.

**Table 1:** Perioperative outcomes of robotic ureteral reconstruction for Endometriosis-Induced strictures

Variable	*N* = 19
Preoperative	
Median Age (IQR), Years	39.0 (30.0-42.5)
Median Body Mass Index (IQR), kilograms/meters^2^	26.1 (20.4–29.7)
Stricture Location	
Middle Ureter (%)	4 (21.1%)
Distal Ureter (%)	15 (78.9%)
Prior Ureteral Stricture Intervention	
Endoscopic (%)	3 (15.8%)
Surgical (Laparoscopic/Robotic/Open) (%)	1 (5.3%)
Preoperative diagnosis of endometriosis (%)	14 (73.6%)
Intraoperative	
Median Operative Time (IQR), Minutes	197.0 (154.0-217.0)
Median Estimated Blood Loss (IQR), Milliliters	50.0 (37.5–55.0)
Median Stricture Length (IQR), Centimeters	2.0 (2.0–3.0)
Robotic Ureteral Reconstruction Performed	
Refluxing Reimplantation (%)	9 (47.4%)
Side-to-Side Reimplantation (%)	4 (21.1%)
Ureteroureterostomy (%)	4 (21.1%)
Buccal Mucosa Graft Ureteroplasty (%)	2 (10.5%)
Median Length of Stay (IQR), Days	1.0 (1.0–1.0)
Postoperative	
New postoperative diagnosis of endometriosis (%)	5 (26.3%)
Median Follow-Up (IQR), Months	22.5 (11.7–41.5)
Major Complications (Clavien > 2) (%)	1 (5.3%)
Surgical Success (%)	18 (94.7%)

Postoperatively, one (5.3%) patient experienced a major (Clavien > 2) complication in which the patient developed an intrabdominal abscess requiring drainage by interventional radiology. The drained fluid was sent for urine creatinine analysis, which returned within normal limits. Five (26.3%) patients were found to have a new postoperative diagnosis of endometriosis based on surgical pathology. At a median follow-up of 22.5 (IQR 11.7–41.5) months, 18 (94.7%) patients were surgically successful. One patient developed a symptomatic recurrence of her distal ureteral stricture 3 months after undergoing a refluxing reimplantation without evidence of residual endometrial implant on cross-sectional radiographic imaging. This patient has been managed with chronic ureteral stent exchanges.

## Discussion

This study presents a multi-institutional experience with RUR for management of ureteral strictures caused by extrinsic compression from endometriotic implants. At a median follow-up of 22.5 (IQR 11.7–41.5) months, we observed a surgical success rate of 94.7%. These findings suggest that RUR techniques can achieve durable outcomes in this complex patient population.

Most of the existing literature centers on ureterolysis as a primary intervention for ureteral obstruction due to endometriosis [6–10]. However, ureterolysis alone may be inadequate for definitive management, especially in cases involving extensive, deeply infiltrative endometriotic lesions. In a prospective review, Mereu et al. evaluated perioperative outcomes of 35 patients who underwent laparoscopic ureterolysis for ureteral endometriosis and demonstrated that 20% of their cohort required a future ureteral reimplant due to persistent ureteral stenosis postoperatively [11]. The authors suggested that ureterolysis should only be favored when there is minimal extrinsic and nonobstructive ureteral involvement [11].

In another study evaluating outcomes of 109 patients who underwent laparoscopic ureterolysis for ureteral endometriosis, 9% of their cohort required reintervention for recurrent ureteral endometriosis at a median follow-up of 52 (IQR 15–109) months [9]. The authors proposed that a greater degree of hydronephrosis and larger endometrial lesions were associated with an increased risk of subsequent ureteral reintervention post-ureterolysis [9]. Other groups have raised concern that dissecting into the adventitia during ureterolysis for endometriosis may risk ureteral devascularization and future stenosis [12].

The surgical approach in our series was individualized based on stricture location, lesion characteristics, and ureteral integrity. The median ureteral stricture length measured intraoperatively was 2 centimeters, reflecting the considerable extent of endometriotic involvement and degree of extrinsic ureteral compression observed in our cohort. In patients with large lesions causing circumferential involvement or necessitating substantial resection of periureteral and adventitial tissue, we performed a transecting ureteroplasty, including a ureteroureterostomy or refluxing reimplantation. Although there are greater concerns regarding the disruption of ureteral vascularity with a transecting approach, it is often necessary in cases requiring extensive resection of diseased periureteral tissue, as it ensures complete removal of the affected ureteral segment. In contrast, in cases where periureteral blood supply may already be compromised, i.e. redo cases, or when resection of the endometriotic lesion did not result in circumferential ureteral disruption, we preferentially employed non-transecting techniques including a side-to-side reimplantation or buccal mucosa graft onlay ureteroplasty. These approaches may help preserve the ureteral blood supply and minimize the risk of ischemia or delayed stricture formation in these settings. Continued refinement and reporting of outcomes will be essential to guide optimal surgical decision-making and utilization of these reconstructive strategies in endometriosis-associated ureteral strictures.

Notably, 26.3% of patients in our cohort had no preoperative diagnosis of endometriosis and were only diagnosed after surgical intervention based on final pathologic analysis. These findings underscore the importance of maintaining a high index of suspicion for endometriosis in premenopausal women presenting with ureteral stricture disease. In several cases, patients may report cyclical pelvic symptoms consistent with endometriosis that had not been elicited during preoperative evaluation. This suggests that in these settings, earlier recognition may be improved through targeted history-taking focused on endometriosis-associated symptoms.

Our study is subject to several limitations. First, its retrospective design and small sample size may introduce selection bias and limit the generalizability of our findings; however, ureteral endometriosis is an uncommon condition, making large-scale prospective studies challenging. Also, we did not assess postoperative fertility or pregnancy outcomes, which may be relevant for certain patients undergoing surgery for endometriosis-related disease. The median age of our cohort was 39.0 (IQR 30.0-42.5) years, suggesting that fertility preservation was likely not a primary concern for most patients. Additionally, surgical decision-making and follow-up protocols varied across participating institutions. Although general management principles were consistent, this inter-institutional variability may limit the standardization of our reported outcomes. Lastly, we did not collect comprehensive follow-up information regarding patients’ concurrent gynecologic management of other endometriotic sites and our ability to assess the durability of ureteral reconstruction in the broader context of disease control is limited.

## Conclusion

A high index of suspicion for endometriosis is warranted in premenopausal women presenting with ureteral stricture disease, particularly in the absence of other identifiable causes. RUR appears to be an effective surgical approach for the management of ureteral strictures secondary to endometriosis. Both transecting and non-transecting approaches are viable options, with selection generally guided intraoperatively by the extent of disease involvement.

## Data Availability

No datasets were generated or analysed during the current study.
